# Southern Surgical and Gynecological Association

**Published:** 1891-12

**Authors:** 


### SOUTHERN SURGICAL AND GYNECOLOGICAL ASSOCIATION.

FOURTH ANNUAL MEETING HELD IN RICHMOND, VA., NOVEMBER 10, 11 AND 12, 1891.

FIRST DAY—MORNING SESSION.

The Association convened in the hall of the Y. M. C. A., and was called to order by the President, Dr. L. S. McMurtry, of Louisville, Ky., at 10 a. m.

Prayer was offered by the Rev. D. M. Hoge.

The first paper read was by Dr. J. W. Long, of Randleman, N. C., entitled "Albuminuria; Its Relation to Surgical Operations." He drew the following conclusions:

1. That neither ether nor chloroform rarely ever injures healthy kidneys.

2. That when renal disturbances from the use of an anaesthetic, the kidneys being healthy, do occur, they are due rather to prolonged narcosis, exposure of the patient, or perhaps to the combined influence of the operation and the anaesthetic.

3. That a mild degree of albuminuria or nephritis, especially if recent, is not a contra-indication to the use of chloroform or ether.

4. That even in the presence of advanced and extensive renal changes, an anaesthetic may be employed, provided the patient and the family are advised of the additional risk.

5. That of the two anaesthetics usually employed, it is yet a mooted question as to which is the safer as far as the kidneys are concerned, unless it be in obstetrical operations.

6. That, while it is by no means the rule, profound functional disturbances and even organic lesions may be induced by an operation, apart from the influence of the anaesthetic.

7. That such renal changes are due to reflex sympathetic action or sepsis, or both.

8. That operations in certain regions, notably the abdominal, genito-urinary, about the mouth and rectum, are specially liable to produce renal complications.

9. That a healthy condition of the kidneys minimizes, but does not obviate, the dangers referred to.

10. That albuminuria is always indicative of renal lesions, and should be regarded with distrust; but is not a positive contra-indication to an operation.

11. That when albuminuria is associated with other evidences of advanced renal changes, no operation should be undertaken without first candidly stating to the patient, or friends, the dangers incident to the condition of the kidneys.

12. That paradoxical as it may seem, an operation will sometimes relieve an albuminuria due to acute affections.

13. That no surgeon is justified in undertaking an operation without first knowing the state of his patient's kidneys.

SYSTEMIC INFECTION FROM GONORRHOEA.

Dr. Bedford Brown, of Alexandria, Va., read a paper on this subject. He cited five interesting cases of systemic infection from gonorrhoea. He believed that there are two channels for the absorption and transmission of the gonorhoeal microbe into the general system. One is by continuity of surface over the mucus membrane of the genito-urinary tract from the urethra to the kidneys. The other chanel is through the medium of the great lymphatic system, from the lymphatics of the urethra to the inguin l glands, thence through the lymphatics of the system, into the general circulation. He believes also that this microbe so transmitted is lodged at different points in the organism. The gonorrhoeal microbe transmitted by continuity of surface over the genito-urinary tract invariably induces specific suppurative inflammation. On the contrary when transmitted through the lymphatics the inflammation is not of a suppurative character, but assumes peculiar types; then the contact of the infectious microbe with the mucous surfaces produces suppurative prostatitis, cystitis, urethritis, pyelitis and then pyo-nephrosis. The absorption of the same through the lymphatic channels first sets up lymphangitis of the lymphatics of the urethra, then lymph-adenitis of Cowper's glands, then of the inguinal glands and inflammation of the connecting lymphatics. By further absorption it may induce septic phlebitis of the thigh, and finally synovitis, endocarditis, and internal destructive ophthalmitis. We also believe that in certain cases genuine septicaemia may be developed in the course of these complications. He thinks there is marked relative difference in the susceptibility of different constitutions to the systemic poisoning of gonorrhoeal infection as in other diseases. That the absorption and infection of the system from this cause is only in exceptional cases. The writer lays stress on gonorrhoeal urethritis following cystitis as a part of the action of the gonorrhoeal infection in its travels over the mucous surface of the genito-urinary organs toward its final destination in this direction, the kidneys. This complication is accompanied with pain at times sharp and paroxysmal, usually dull and aching in character. These sharp paroxysms of pain extend upward to the kidneys and not downward to the bladder as in nephritic colic. Then again, there is soreness in the entire line of the urethra, increased on pressure, so that the course of the canal may be marked out clearly. Urethritis is always established before nephritis begins in gonorrhoeal infection.

The cases cited by Dr. Brown indicate that a state of pyaemia or septicaemia may be developed by systemic infection from gonorrhoea in certain cases.

Dr. J. Edwin Michael, of Baltimore, Md., read a paper entitled :

A REPORT OF SOME ADDITIONAL CASES OF EXTERNAL PERINEAL URETHROTOMY WITHOUT A GUIDE, in which he said that the operation is one of great value both in gonorrhoeal and traumatic cases, and he thinks one is justified in bringing forward any experience in it which may be of use to the profession. His results were very satisfactory, a fact which he attributes rather to the fortunate circumstance that his patients were largely free from grave constitutional disease than to any method or application which he had to suggest. He had simply followed what he considered the precepts of good surgery as applied to this region of the body, viz., free incision, free drainage, and as much of antiseptic surgery as the circumstances would allow.

The report of these eight cases brings up the number of patients on whom he made the operation of external perineal urethrotomy to seventeen, and the conditions which made the operation necessary include nearly all those which ordinarily indicate it. None of the patients have died at a period nearer than six months to the time of the operation, and the deaths which have occurred were due to other causes. In the Spring of 1887 he reported nine cases of perineal section without a guide, and he had only to add to the remarks made at that time, that increasing experience leads him to have more and more confidence in the good results of opening the perineum and less fear of danger.

It is true that he had had unusual good fortune in operating on cases which as a rule presented no grave kidney lesion, but while it must be admitted tha[illegible] such complication adds to the risk of the operation as much if not more than it does to others of equal gravity, he is firmly convinced that opening the perineum in old stricture cases with bad kidneys is much freer from danger than internal urethrotomy or even dilatation. A case in point. About ten years ago he did an internal urethrotomy on a patient with an old tough stricture. In forty eight hours he had a temperature of 107 degrees, and was very ill. The same patient returned to him a short time since. He could pass a No. 10 E. sound with difficulty. The stricture was resilent and closed after the sound to such an extent that urination was difficult and unsatisfactory. The patient had been having chills, and was somewhat nauseated and weak. His urine, although ammoniacal and ropy, contained no evidence of grave kidney trouble. He proposed a combined internal and external urethrotomy and refused to do either operation without the other. The patient consented. He opened the perineum freely and cut the urethra with the Otis instrument to 40 F. The temperature did not rise above 100 degrees, the patient did well in every particular, and in three weeks he sent him home passing a No. 36 F. instrument on himself.

### FIRST DAY—AFTERNOON SESSION.

Dr. W. F. Westmoreland, of Atlanta, Ga., read a paper entitled "Reduction of Dislocations by Manipulation."

Dr. Joseph Price, of Philadelphia, Pa., followed with a paper, "Complications in Pelvic Surgery, and How to Deal With Them." The author's reasons for choosing this subject, were that the importance of recognizing the part that complications play in the work of the surgeon, are not appreciated by the generality of medical men, by general surgeons, and least of all, by the tyro in surgery, and by those who are anxious to be gin their surgical investigations and trial trips by an entrance in to the domain of abdominal or pelvic surgery. The complications in this special branch of surgery are primarily those of surgery in general, with many things superadded to render them formidable. It may be the intention of the surgeon to remove the appendages for a bleeding fibroid. In ordinary operations the removal of the uterine appendages is to the skilled abdominal or pelvic surgeon one of the simplest of undertakings. If, however, he attempts to accomplish their removal, without holding in mind the complications that as a rule exist, or if he is a neophyte or an experimental dabbler, he will find too late in many cases, that he has attempted an operation he cannot finish, or if he does complete it, he has also sacrificed his patient, or rendered her worse off than before. In other words, to accomplish a cure, he must abandon removal of the appendages and perform hysterectomy, which has but little in common with the operation originally proposed. If this idea is still further carried out, we shall find that complications do not confine themselves to one system of organs, but extend to all surrounding structures by reason o inflammatory adhesions. This is true of the bladder, uterus, intestines, omentum, stomach and liver. Adhesions are the bane of abdominal and pelvic surgery, and hence we see that the greatest mistakes and failures are made by those who from a knowledge of abdominal surgery simply have attempted to deal with pelvic inflammations. The abdominal surgeons who can be counted as really successful pelvic surgeons are therefore few. This is said with no intention of detracting from the importance of abdominal surgery. The strictly abdominal organs must always enter largely into the domain of surgery.

With regard to irrigation, we must get out of our heads the idea that it is dangerous. Too often in the writer's experience has hot water brought about a speedy reaction in patients whose lives were almost despaired of. We are told that cases do not need flushing, that they do badly under it. Dr. Price believes that they do need flushing if they are desperate cases, and if they do badly they do so, not on account of the flushing, but because of the operation that preceded it. Next we have a resort in packing. Gauze packing accurately applied to the bleeding or oozing surfaces, so that it can be removed without interfering with the otherwise completed operation, is of infinite value in hemorrhage. It can be suffered to remain indefinitely almost, broadly speaking—at least up to sixty or seventy hours, if absolutely clean and fresh, either salicylated or iodoformized. The drainage tube controls hemorrhage. The drainage tube is currently spoken of as if it were an annex to pelvic surgery, easily dispensed with. The writer uses it almost without exception in adhesions. His results are better than those obtained without its use.

The plea of the paper was for absolutely exact painstaking work, that shall leave nothing for regret, nothing to do over, nothing to explain, but shall stand out in the light of results as justifiable, scientific and perfect, when put beside methods that palliate without curing, and are no more a part of real surgery than is hypnotism refreshing sleep.

"Laparotomies Performed During the Past Year." This was the title of a paper read by Dr. Thomas Opie, of Baltimore, Md.

The tabulated statement accompanying the paper embraced thirty-two abdominal sections made in the twelve months beginning November 1, 1890, and ending October 31, 1891. The operations were performed consecutively as set forth in the accompanying table. They were

| | |
|---|---|
| Ovarian tumors, | 6 |
| Chronic ovaritis, | 7 |
| Fibroid tumors, | 4 |
| Pyosalpinx, | 5 |
| Retroflection with adhesion and dysmenorrhoea, | 3 |
| Exploratory incisions, | 3 |
| Extra-uterine pregnancy, | 1 |
| Abcess of ovary, | 1 |
| Cyst of broad ligament, | 1 |
| Cystic degeneration of ovary, | 1 |
| | 32. |

Nine of these patients were operated on in the amphitheater before the whole class at the College of Physicians and Surgeons, The remainder, twenty three, were operated on privately. Twenty seven were white, and five were colored. The deaths were as follows: Ocphorectomy for pyosalpinx, 1; shock from ovariotomy, 1; oophorectomy for acute mania, 1; and abdominal hysterectomy for fibro-cystic tumor, 1. Total 4.

*Stitch abscess.*—This complication occurred nine times, a much larger number relatively than he had seen recorded heretofcre, while no case proved disastrous, several were exceedingly annoying in delaying patients in hospitals. They occur most frequently in cases where the drainage tube has been used. The early opening of the abdominal dressings for any purpose favor their occurrence. When the dressings remained intact for seven days there seemed to be the greatest immunity from the stitch abscess.

Drainage was resorted to in but three cases during the year. Case 11, ovarian and dermoid cyst, had a drainage tube in five or six days, and the writer is convinced that it retarded the patients' convalescence. He is of the opion that too much flushing is done; that it is but seldom called for. A plentiful supply of fine, properly prepared, elephant ear sponges will do away with flushing in most cases and remove the necessity for drainage. They are efficient helps in keeping the abdomen free from infection.

Dr. Cornelius Kolloch of Cheraw, S. C., read a paper entitled "Ovarian Cysts, With the Report of a Case of Ovariotomy of a Young Girl."

He said the causes of ovarian cyst seemed to be still a question *sub judice* in the minds of those who are most progressive and who have made the greatest advancement in the science of gynecology. Various theories have been put forth by those of larger experience, who are earnest seekers after truth, and who are patient investigators of all unnatural and morbid phenomena. But no satisfactory decision has been obtained from all the patient and searching investigations that have been made as to the cause of this singular, unaccountable and sometimes fatal neoplasm, characterized by histological diversity from the viscus of which it is a production. Some of the theories seemed, at a glance, to be plausible, but upon close study we find that they will not bear inspection.

CASE.—Miss C. L. H., aged 11 years, 8 months and 19 days; general health perfect in every particular. Menstruation first appeared about two months before she was 11 years of age, continued with perfect regularity, never excessive or scant, nor was it eccompanied by the slightest pain. Her physique was fine in every way. Though less than twelve years of age, she weighed 135 pounds; was strong and active. Her breasts were as full and large as those of a woman at 35. She was very handsome, had a fine voice and sang beautifully. She was very intellectual and stood at the head in all her classes in a large high school. He saw her for the first time on the 9th of January, 1891. The abdomen was greatly distended, but facies ovariana was not very pronounced. He was confident she had an ovarian cyst, and he rather suspected she had two. On the 16th of January he made a section about three inches below the umbilicus and removed a cyst from each side, the one on the left weighing twelve pounds and that on the right seven pounds. A more prompt and complete recovery the writer had never seen from the simplest operation. Union by first intention took place and the sutures, silver wire, were removed at the end of the seventh day. In twelve days she was up and about her room and on the 23rd day after the operation returned to her home, a distance of 200 miles

It is now ten months since double ovariotomy was done.on this young girl, and there has not been the slightest discharge from her of any kind. At each menstrual period there is considerable commotion in the pelvic region, attended with some uneasiness in the head and back, but at each period these symptoms decreased and the last two were accompanied by no pain whatever. The remarkable physical development in this case still continues. It is now ten months since the operation. She has regained six pounds in weight, weighs 141 pounds and looks better than before she underwent ovariotomy. This young girl came from the purest and healthiest stock of people in this region. Not an individual on either side was ever known to have any constitutional trouble of any kind. Her mother and family physician, both highly intelligent, say they never knew her to be the least indisposed in any way.

(Continued in our next issue).

